# New Graphene Composites for Power Engineering

**DOI:** 10.3390/ma15030715

**Published:** 2022-01-18

**Authors:** Tadeusz Knych, Andrzej Mamala, Paweł Kwaśniewski, Grzegorz Kiesiewicz, Beata Smyrak, Marek Gniełczyk, Artur Kawecki, Kinga Korzeń, Eliza Sieja-Smaga

**Affiliations:** Non-Ferrous Metals Faculty, AGH University of Science and Technology, 30-059 Krakow, Poland; tknych@agh.edu.pl (T.K.); amamala@agh.edu.pl (A.M.); kwas@agh.edu.pl (P.K.); gk@agh.edu.pl (G.K.); mgmarkoni@gmail.com (M.G.); akawecki@agh.edu.pl (A.K.); kkorzen@agh.edu.pl (K.K.); esmaga@agh.edu.pl (E.S.-S.)

**Keywords:** graphene, copper, aluminum, overhead conductors, composite

## Abstract

Intensive research is underway worldwide to develop new conductive materials for applications in the power industry. Such tests aim to increase the electrical conductivity of materials for conductors and cables, thus increasing the current carrying capacity of the line and reducing the loss of electricity transmission. The scientific discovery of recent years, graphene, one of the allotropic types of carbon with very high electrical and thermal conductivity and mechanical strength, creates great opportunities for designing and producing new materials with above-standard operational properties. This project concentrates on developing technology for manufacturing aluminum-graphene and copper-graphene composites intended to be used to produce a new generation of power engineering conductors. In particular, we present the results of the research on the mechanical synthesis of aluminum-graphene and copper -graphene composites, as well as the results of the electric, mechanical, and structural properties of rods obtained after the extrusion process and wires after the drawing process.

## 1. Introduction

Copper has high electrical conductivity (58 MS/m at 20 °C) and is widely used in electrical applications. Owing to a favorable ratio of mechanical strength to mass density and resistivity, aluminum is a conductive material competitive for copper and commonly used in power engineering, in particular in overhead power lines as ACSR (Aluminium Conductor Steel Reinforced) cables. 

Continuously growing demand for higher transmission capacity of power lines is a driving force of the development of conductive materials engineering. Operating conditions of conductors impose additional requirements on conductive materials concerning strength and fatigue characteristics, rheology, and excellent corrosion resistance. It limits the application of pure metals, i.e., pure copper and pure aluminum, to produce power conductors and fosters the use of alloys that demonstrate high strength and are resistant to operating conditions. Consequently, electrical conductivity is reduced. 

One of the dynamically developing directions of improving electric current transmission is superconductivity. Actually, the zero resistance, and consequently the lack of dissipation of electrical energy, mean superconductors are commonly used in the windings of superconducting electromagnets (e.g., in medicine, computed tomography, physics). The race to discover materials with an increasingly higher critical temperature, exceeding 100 K, is still ongoing, e.g., 1988-YBaCuO (Tc ≈ 100 K), 1995-HGBaCaCuO @ 30 GPa (Tc ≈ 160 K, 2000-MgB_2_ (Tc ≈ 40 K), 2010-SrFFeAs (Tc ≈60 K), 2010-FeS (Tc ≈ 100 K) and 2015-H_2_S @ 155 GPa (Tc ≈ 200 K). One of the latest discoveries is a H_2_S superconducting material that obtains superconducting properties at a temperature of 200 K but at a high pressure of 155 GPa. Extremely low temperatures are no longer a requirement to generate superconductivity, and the discovery of superconducting materials operating at close to room temperature could find application in, for example, current transmission technology, where losses due to resistance in transmission lines could be almost completely avoided [1,2]. Another direction of research for improving the capacity of industrial lines is graphene. Graphene is a two-dimensional carbon material that has attracted great scientific and technological interest due to its intriguing physical properties and enormous potential for various applications [3,4,5]. All this has led to the development of rapidly evolving research within the field of graphene, based on the deposition of high quality and uniform thin films with controllable thickness over large areas. 

Graphene has recently become a very attractive alternative as a new kind of “alloy additive” to copper and aluminum, allowing modification of their electrical and working characteristics. Unfortunately, concerning the low solubility of carbon in the metals, graphene is mainly used as a composite additive. Another mechanism of changing the electrical characteristics of the materials involves the formation of metal-carbon bonds called Covetic. Moreover, very high mechanical strength and the high melting point of graphene can positively affect the composite characteristics, increasing its resistance to operating conditions, including long-term temperature exposure—the parameter is closely related to the current-carrying capacity of power conductors.

Developing an effective graphene production technology and discovering its characteristics created new possibilities for making materials with non-standard characteristics. The greatest expectations concern the non-standard specific electrical conductivity of the materials. Table 1 presents selected physical characteristics of copper, graphene, and nanotubes. Special attention should be paid to very high specific electric conductivity, low weight, and high mechanical strength of carbon materials compared to copper and aluminum. Many centers in the world have recently performed intense conceptual works and studies on graphene/nanotube synthesis with copper and other metals to change their physical characteristics and improve their functional characteristics [6,7,8,9,10]. 

Nowadays, works are conducted globally towards implementing new solutions using composites of metals and different allotropic forms of carbon. 

Special aluminum composites—carbon fibers—are used for carrier and conduction cores of power conductors. It helps improve the transmission capacity of conductors and reduce their weight and sagging, which are among the most important operating parameters of power lines. The Naval Research Laboratory in the USA developed high-resistance graphitized carbon fibers with excellent electrical characteristics. Works on copper-carbon fiber composites with improved mechanical strength are in progress. Nanotubes are another form of carbon used in power engineering. In 2010, in the USA, prof. Chen patented a Cu-CNT composite with almost 40% higher conductivity than copper [11]. Prof. Castro made Al-CNT wires with ca. 30% higher conductivity than pure aluminum. Currently, leading global centers are working towards the development of such solutions. An advantage of graphene, which makes it stand out among other allotropic forms of carbon, is that it is not prone to form agglomerates (as nanotubes are) and has a much greater active blending area with the matrix than nanotubes. Moreover, global trends related to the application of carbon bring newer and cheaper technologies of acquiring this type of raw material. To that end, leading research and implementation centers work on developing methods of manufacturing and processing aluminum-graphene or copper-graphene composites. Such works are conducted in the following centers: US Army Benét Laboratories, Armaments Research Development and Engineering Center, Watervliet, Rice University, and Rensselaer Polytechnic Institute in the USA. Nowadays, the only application of graphene prepared for common use in the near future is in touch screens developed by the Korean Samsung [12,13,14,15,16,17,18,19].

Synthesis of graphene with aluminum or copper creates an opportunity to obtain new materials with previously unknown electrical and functional characteristics. There are different approaches and works on using different methods to develop such materials are in progress and include the following:

(a) Chemical synthesis—Covetic types of an atomic blend (blending by synthesis of liquid metal with graphene, under appropriate casting conditions) [20,21].
(b) Electrochemical deposition of graphene on elements of copper or another material [22].
(c) Casting a mixture of liquid metal with graphene to a form facilitating further plastic processing to make wires with required characteristics (continuous-cast conductors as input material for further wire making can be the product of the process).
(d) Deposition of monoatomic carbon coats (graphene) using the CVD method on the surface of Al or Cu wires and their further plastic processing to form wires with required characteristics.
(e) Mechanical synthesis of Al powders coated with graphene or Cu powders coated with graphene to a form facilitating their further plastic processing to make wires with required characteristics [23,24,25,26,27,28,29,30].

There is a lot of research in the literature on the synthesis of graphene from metals. Authors of papers [20,21] presented detailed analysis development of copper covetic. Copper covetic is a material consisting of a copper matrix reinforced with the second phase, which is a chemical compound of carbon and copper forming a covalent bond between carbon and copper. It is assumed that the second phase is extremely fine-dispersive and is of nanometer dimensions. An advantage of such a bond is that it does not cleave during the material re-melting. This material exhibits excellent functional properties: a 50% increase in tensile strength, a 50% increase in thermal conductivity, and a 70% increase in electrical conductivity and fuse current over pure copper [20,21]. Wang et al. [31,32] presented the research results on the synthesis of the Al-C composite with a content of 0.3% wt. graphene. The addition of graphene led to an increase in the strength of the composite to the value of 249 MPa, which was an increase of 62% compared to pure aluminum (154 MPa). The authors of the work to produce the above-mentioned composite used a spherical aluminum powder ball milling process. To improve wettability, they used a 3% water solution of polyvinyl alcohol (PVA), thanks to which a hydrophilic membrane was formed on the surface of the aluminum particles. The obtained GO/Al composite powders were heat-soaked at 550 °C in an argon protective atmosphere for 2 h in order to decompose the PVA and reduce GO to a graphene nanosheets (GNS) sheet. The densification and consolidation of the powders consisted of pressing the powders, sintering at 580 °C/2 h, and hot extrusion at a temperature of 440 °C. Interesting results of the synthesis of graphene with metals using HPT (High-Pressure Torsion), a technique involving the simultaneous twisting and squeezing of the material under high pressure, are presented in the article [33] by Y.Huang, P.Bazarnik, M.Lewandowska, and others. Researches produced nanocomposites by combining aluminum with graphene in the amount of 5%, by mixing and pressing them at room temperature, and then by the HPT process in different temperatures (100 and 200 °C) in different torsional stresses and numbers of twists was made. Most of the methods used to synthesize aluminum-carbon composites involve high-temperature processes, which may directly influence the oxidation of the metal matrix or the reactions that occur at the metal-carbon interface. The applied HPT method allows for strong plastic deformation, perfecting the grain and, as a result, improving its mechanical properties. Composites aired with this method obtained tensile strength at the level of 350, 340, and 290 MPa and electrical conductivity at 25 °C at the level of approx. 65% IACS—i.e., higher than pure aluminum. 

Literature analysis shows that there are many methods of synthesizing aluminum and copper with graphene. It is difficult to say which one guarantees composites with the properties required for electrical applications, i.e., high electrical conductivity and high tensile strength. This is due to the problems associated with the poor wettability of graphene and copper or graphite and aluminum, the large difference in the density of copper and graphene, and the unstable quality of graphene currently available on the market [34,35,36,37,38].

## 2. Materials and Methods

The purpose of the study was to evaluate the synthesis of copper and graphene as well as aluminum and graphene and the formability of composites during the extrusion process. The tests involved the implementation of a powder synthesis of copper and aluminum powders with graphene. 

### 2.1. Raw Materials for Synthesis 

Mixtures of appropriate ratios of copper and aluminum powders and graphene were used to synthesize the powders. Graphene was produced in the Institute of Electronic Materials Technology in Warsaw within a project called “New wires made of metal-graphene composites with improved conductivity, intended for power conductors”, implemented as a part of the Applied Research Programme by METGRAF Consortium. The Raman spectrum studies were performed with Renishaw inVia Confocal Raman Microscope – parameters: excitation (cw): lasers 532 nm, 633 nm, 1064 nm, 325 nm, configuration: back-scattering, spatial resolution: 0.5 microns, resolution: about 1.5 cm^−1^, spectral range (Raman shift): 50 cm^−1^–6000 cm^−1^, experimental temperature: ambient.

**Figure 1:**
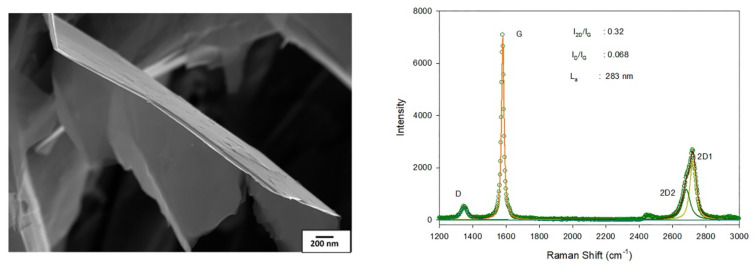
Flaked graphene, SEM, magnification: 25,000× (**left**), Raman spectrum (**right**).

Figure 1 presents the results of SEM analysis and Raman spectrum of graphene used in the studies. Based on the analysis of the Raman spectrum, it was possible to determine the degree of deterioration of the produced layer, the stress of the layer, and the estimation of the number of layers. From the Raman spectrum (from the position of the 2D peak and its width, as well as from the ratio of the intensity of the 2D peaks to G), it can be concluded that the sample consisted mainly of graphite flakes with a size of about 280 nm, covered with a thin layer of graphene.

**Figure 2:**
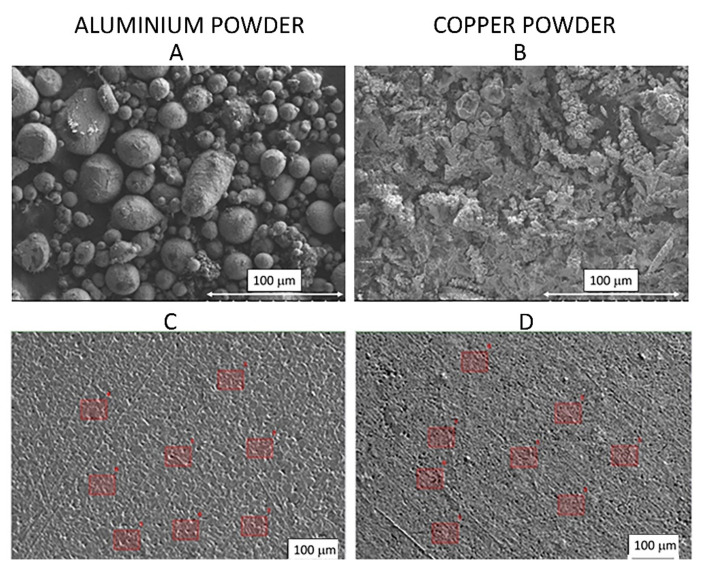
Aluminum and copper powder SEM images magnification × 500 ((**A**)—Al powder, (**B**)—Cu powder) and compressed Al and Cu powder with selected points for chemical analysis by WDS (images (**C**,**D**)).

Figure 2 shows the results of SEM analysis of aluminum and copper powders (A and B) and selected points for chemical composition analysis by WDS method, compressed powder sample (C and D), single measurement area: 60 µm × 45 µm.

The chemical composition of Al and Cu powders selected for synthesis was tested on the JXA 8230 microanalyzer by Jeol (Tokyo, Japan). An accelerating voltage of 15 kV was used, the electron beam current was 30 nA. The measurements were carried out using waveguide spectrometers (WDS, Oxford Instruments, High Wycombe, UK). The wavelength dispersion method enables a more accurate measurement of light elements. It is also characterized by a better detectability of elements and a higher resolution of spectral lines. Table 2 shows the results of the chemical composition analysis of powders selected for mechanical synthesis with graphene.

### 2.2. Synthesis Parameters

**Figure 3:**
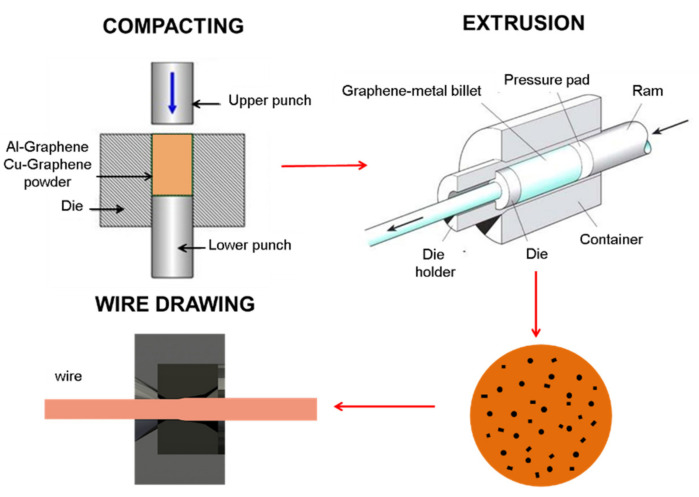
Diagram of wire production from Cu-C and Al-C composites (compaction, extrusion, and wiredrawing).

The synthesis involved mixing and compaction copper powders and aluminum powders with graphene, pressing them into compacts, and their further consolidation using the conventional extrusion method. The powder mixtures forming the Al-C and Cu-C composites were made according to the proportion of graphene addition: 0.5% and 1% by weight based on the weight of pure aluminum/copper powder. The mixing process was carried out using industrial and laboratory ball mills for 30 to 60 min. The diagram of the composites and wires production process is shown in Figure 3.

**Figure 4:**
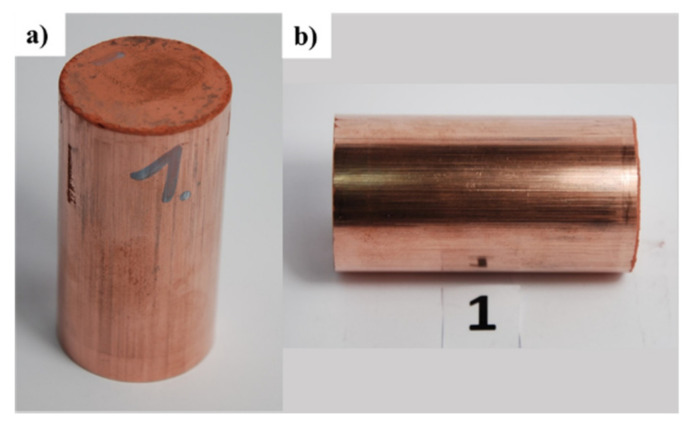
Copper-graphene composites (CUGRAF) obtained by the consolidation of powders: (**a**) view of the cross-sectional surface, (**b**) view of the lateral surface.

**Figure 5:**
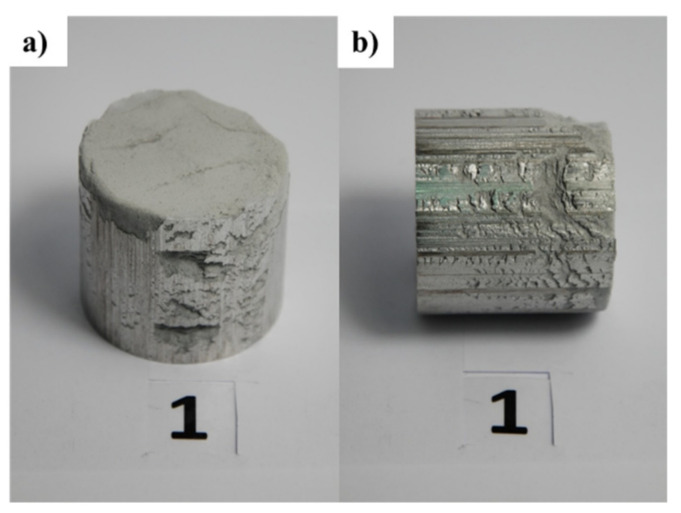
Aluminum-graphene (ALGRAF) obtained by the consolidation of powders (**a**) view of the cross-sectional surface, (**b**) view of the lateral surface).

The criterion for selecting the mixing time of the powders was related to obtaining a homogeneous distribution of graphene in the volume of the metal matrix. Table 3 presents material parameters (graphene content in Cu-C and Al-C composites) and parameters of consolidating copper and aluminum powders with graphene (mixing process parameters and consolidation conditions). Figure 4 and Figure 5 present composites of copper and aluminum powders with different graphene content produced using the method mentioned above.

In the case of ALGRAF composites, the increase in graphene content led to a deterioration of the compacting effect, which resulted in the production of moldings and presses with poor surface quality (see Figure 5). 

Composites thus made were extruded using a special stand horizontal press with a rotating die. In the next step, extruded rods with a diameter of 4 mm were strained in 98% (strain hardening) by the wire drawing process, which helped obtain wires with a diameter of 1 mm. Detailed parameters of the extrusion and wires drawing process are presented in Table 4. 

### 2.3. Methods of Testing the Properties of Composites

Wires after the drawing process and rods after the extrusion process were tested for their mechanical, electrical, and structural properties. The tests of mechanical properties were carried out using the uniaxial tensile test. The mechanical properties of the samples were measured on a Zwick/Roell testing machine (Zwick, Ulm, Germany) with a maximum force range of 20 kN. The tests of electric properties were performed using the Thomson bridge. The maximum measuring resolution was 1 nΩ, with measuring currents from 100 μ to 10 A. The accuracy class was 0.01%. Microstructure tests were performed using a Scanning Electron Microscopy (Hitachi Model, Kasama, Japan) and Jeol JXA 8230 microanalyzer (EDS analysis). An accelerating voltage of 15 kV was used, the electron beam current was 30 nA. 

## 3. Results and Discussion

The main objective of the research was to answer whether the Al-Graphene and Cu-Graphene composites produced a guaranteed homogeneous structure and chemical composition throughout the entire volume of the composite. 

**Figure 6:**
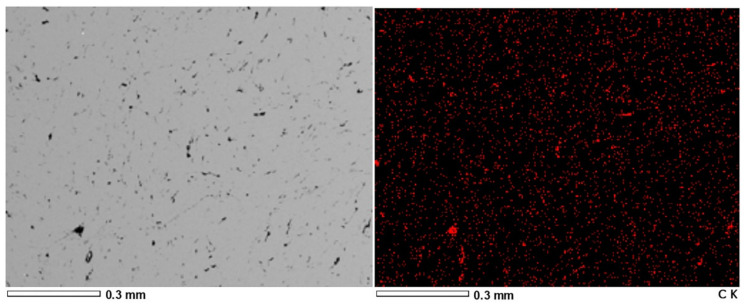
EDS analysis of graphene distribution in sample no 1—rods after the extrusion of CUGRAF composites (1% by weight of graphene).

**Figure 7:**
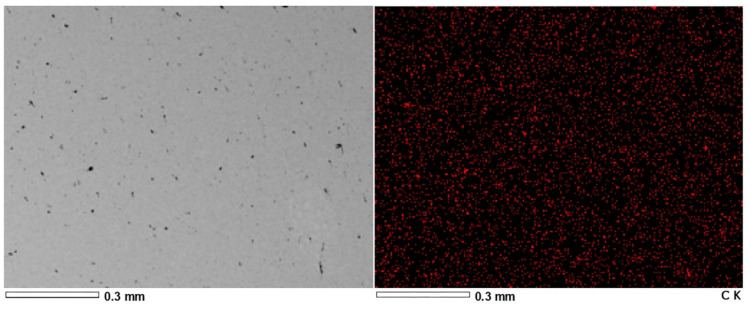
EDS analysis of graphene distribution in sample no 3 (rods after the extrusion of ALGRAF composites—1% by weight of graphene.

Figure 6 and Figure 7 present the results of EDS analysis of graphene decomposition in the matrix of copper and aluminum extruded bars with a graphene content of 1% by weight.

Based on the research, it was found that the composites obtained both as a result of plastic consolidation (extrusion process) of ALGRAF and CUGRAF showed a homogeneous distribution of graphene in the entire volume, which guarantees a homogeneous distribution of properties along the length of the wires produced.

Table 5 shows the results of the tests of mechanical and electrical properties produced for further processing. 

One can observe that the higher content of graphene in CUGRAF and ALGRAF composites significantly reduced the plasticity of the material. Nevertheless, no significant differentiation of the tensile strength was observed, and the values of 200–250 MP (for Cu-C) and 140–150 MPa (Al-C) for the majority of materials were reached. An analysis of the impact of graphene content on electrical properties of the studied composites at different processing stages revealed slightly higher resistivity values as compared to the base material, i.e., pure copper and pure aluminum. This was the evidence of the probable presence of graphene material in a form not linked with the copper or aluminum matrix. Additionally, the lack of reduction in resistivity could result from imperfect consolidation and processing of the composites.

**Figure 8:**
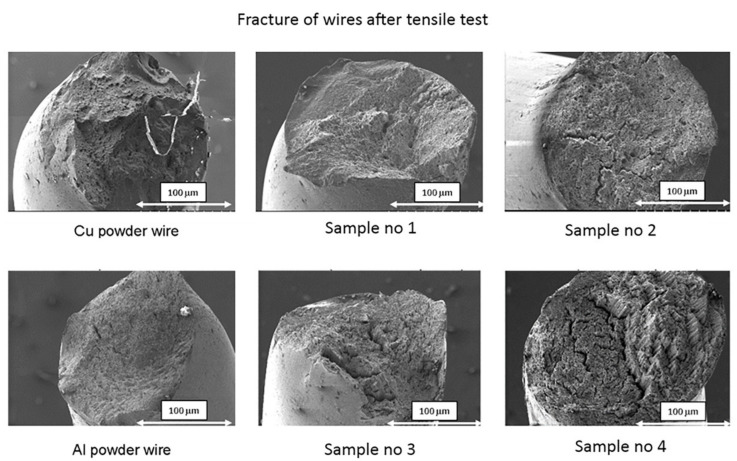
SEM image of wires fracture after tensile test according to Table 6, magnification × 100.

After the tensile tests, wire fracture was analyzed by SEM. Photos of fractures of wires made of ALGRAF and CUGRAF composite are presented in Figure 8. The shape of the fractures in practically all cases is similar to the brittle fractures. There are no plastically deformed areas. The general shape of the fractures in all cases is similar to the brittle fractures. There are no plastically deformed areas—typical for aluminum and copper wires. A detailed analysis showed delamination of the material in the case of ALGRAF composite wires with the highest graphene content. The above phenomenon may result from the lack of good wettability of graphene to aluminum and, consequently, the lack of a strong bond of graphene with the aluminum matrix.

**Figure 9:**
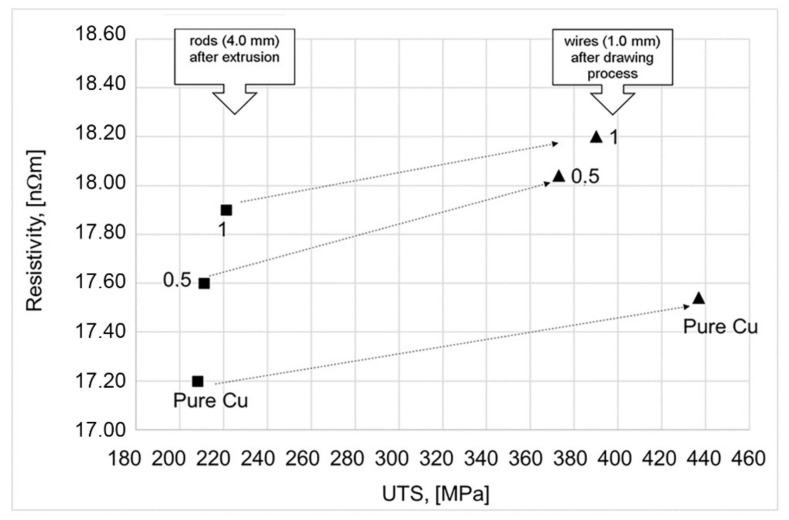
Relationship between UTS and resistivity of CUGRAF composite after extrusion and after wire drawing process (sample no 1 and 2 and pure Cu).

**Figure 10:**
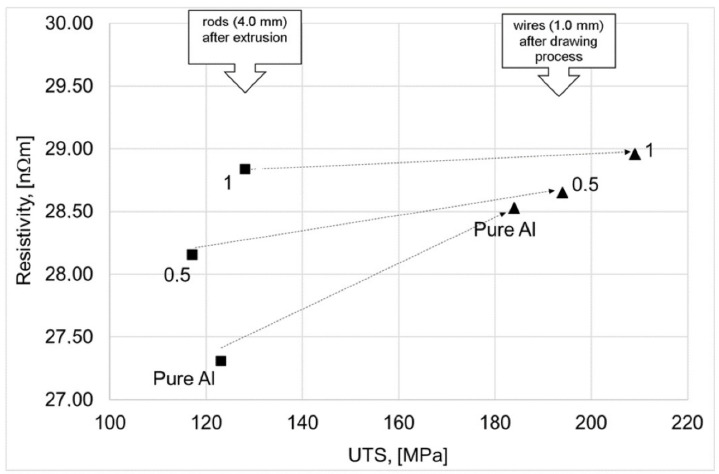
Relationship between UTS and resistivity of ALGRAF composite after extrusion and after wire drawing process (samples no 3 and 4 and pure Al).

Figure 9 and Figure 10 show graphs illustrating the relationship between the (UTS—Ultimate Tensile Strength) and resistivity of the rods obtained in the extrusion process and wires obtained in the wiredrawing process. 

Based on the relations presented in Figure 9 and Figure 10, it was found that the differentiation of the graphene content in copper leads to different UTS values. It was found the above relationship is close to linear. The highest increase in UTS was found in composites of pure Al and Cu powders, and the lowest in composites containing 1% graphene.

On the other hand, in the case of extruded rods, it has been observed that the addition of graphene to the Al matrix and Cu matrix leads to an increase in resistivity (a decrease in electrical conductivity). Interestingly, in the case of aluminum-graphene rods and wires, the differentiation of the resistivity value was concluded for various graphene content. Higher resistivity values of wires compared to rods are an effect of dislocation density increasing. While similar Al-C wires resistivity values—regardless of the graphene content—results from the increase in their density, an additional effect was compacting due to cold deformation (the deformation value is at the level of 96%). In the case of copper-graphene composites, this situation does not exist. The differentiation of the resistivity value for Cu-C rods after extrusion and for wires after the drawing process is similar.

The wires obtained by the laboratory drawing process did not show any lower than standard resistivity at room temperature compared to the reference materials (Cu-ETP or pure Al.—see Table 1). The lack of a decrease in the electrical resistivity of composites is related to the high resistivity (to low electrical conductivity) of the graphene used and the synthesis method. One of the major difficulties in the mechanical synthesis of Cu-C and Al-C composites is the poor wettability of copper and aluminum with graphene. Perhaps using a sintering process would make it possible to obtain better results related to the electrical properties.

## 4. Conclusions

Based on the results of experimental research, it was found that:

1. As a result of the mechanical synthesis process consisting of mixing and compacting (without sintering), Al-C and Cu-C composites with homogeneous carbon distribution were obtained.
2. The results of the drawing process showed good deformability of composite wires in the drawing process. At the same time, a limited ductility was found, which is expressed by a lower total deformability of these materials compared to traditional materials. The above fact results from the significantly lower plasticity of composite wires.
3. The addition of graphene to aluminum and copper results in an increase in resistivity and strength
4. The addition of graphene to copper and aluminum results in a different type of strain hardening during the wiredrawing process. The addition of graphene to aluminum leads to greater strain hardening (increasing UTS: pure Al—49.6%, Al-C0.5% wt.—65.8%, Al-C1% wt.—63.3%). On the other hand, in the case of Cu-C composites, the addition of graphene resulted in a lower strain hardening in the drawing process (increasing of UTS: pure Cu—110.1%, Cu-C0.5% wt.—76.8%, Al-C1% wt.—76.5%). The different types of strain hardening result from a different mechanism of graphene interaction on Cu and Al.
5. The obtained results showed that it is possible to produce wires from composites based on aluminum/copper powders with the addition of graphene in the core of the wires, which can be a component of overhead power cables.

## Figures and Tables

**Table 1:** Selected properties of copper, aluminum, and graphene [6,7,8,9,10,11,12].

Parameter	Traditional Materials		Modern Materials	
	Aluminium	Copper	Nanotubes	Graphene
Melting point [°C]	660	1083	4520	4620
Density [g/cm^3^]	2.76	8.92	1.3–1.4	1.1–1.5
Mobility of electrons, [cm^2^ v^−1^ s^−1^]	1.2	4.33	10,000	15,000
Conductivity [MS/m]	36	58	280 (1.5–5 × Al)	100 (3 × Al)
Thermal conductivity [W/m^.^k]	200	400	3500	4840^±440^–5300^±480^
Tensile strength [MPa]	60–200	200–400	11,000–63,000	130,000
Temperature coefficient of resistance [K^−1^]	4.0 × 10^−3^	3.9 × 10^−3^	-	-
Young Modulus [GPa]	70	120	1000	1000
Atomic radius [pm]	125	128	70	70
Lattice parameter [pm]	404	360	142	142

**Table 2:** Chemical composition results of aluminum and copper powders (WDS method).

Measuring	Al. Powder					Cu. Powder					
	Chemical Composition, %wt.					Measuring	Chemical Composition, %wt.				
Point No	O_2_	Cr	S	Fe	Al	Point No	O_2_	Cr	S	Fe	Cu
1	0.819	0.024	0.003	0.118	98.4	1	0.355	0.000	0.006	0.000	99.2
2	0.743	0.018	0.007	0.138	98.2	2	0.311	0.000	0.012	0.013	99.17
3	0.868	0.000	0.004	0.11	98.19	3	0.296	0.034	0.000	0.000	99.07
4	0.846	0.000	0.000	0.112	98.45	4	0.295	0.013	0.007	0.006	99.18
5	0.721	0.000	0.000	0.127	98.43	5	0.24	0.003	0.000	0.000	99.42
6	0.779	0.000	0.000	0.108	98.46	6	0.256	0.012	0.000	0.000	99.42
7	0.876	0.011	0.005	0.114	98.53	7	0.234	0.000	0.000	0.028	99.28
8	0.745	0.000	0.002	0.117	98.40	8	0.266	0.000	0.012	0.017	99.18
Average	0.800	0.007	0.003	0.118	98.38	Average	0.282	0.008	0.005	0.008	99.24

**Table 3:** Synthesis parameters of Cu-C and Al-C composites with different graphene content.

Type of Composite	Milling					Consolidation	
	Sample No	Powder Mass (g)	Graphene Mass (g)	Graphene Content		Milling Time	Pressure
				%wt.	%vol.	min	atm
Cu-C	1	500	2.5	0.5	0.6	30–60	30–50
Cu-C	2	500	5	1	1.2	30–60	30–50
Al-C	3	100	0.5	0.5	0.6	30–60	30–50
Al-C	4	100	1	1	1.2	30–60	30–50

**Table 4:** Consolidation process parameters of CUGRAF and ALGRAF composites with different graphene content.

Type of Composite	Extrusion Process		Diameter Rods after Extrusion, (mm)	Diameter Wires after Drawing Process, (mm)
	Temperature, (°C)	Force, (kN)		
ALGRAF (40 mm)	200–400	800	4	2
CUGRAF (40 mm)	400–600	1000	4	2

**Table 5:** Mechanical and electrical properties of the CUGRAF and ALGRAF rods after the extrusion process.

Sample No	Ultimate Tensile Strength (UTS), (MPa)	Yield Stress (YS), (MPa)	A_100_ (%)	Resistivity 20 °C (nΩm)
1	211	158	11.3	17.6
2	221	162	10.2	17.9
3	117	98	3.1	28.16
4	128	114	3.2	28.84
Pure Cu	208	154	25.0	17.2
Pure Al	123	100	5.7	27.31

**Table 6:** Mechanical and electrical properties of the CUGRAF and ALGRAF wires after the drawing process.

Sample No	Ultimate Tensile Strength (UTS), (MPa)	Yield Stress (YS), (MPa)	A_100_ (%)	Resistivity 20 °C (nΩm)
1	373	363	2.20	18.04
2	390	376	2.60	18.20
3	194	187	1.52	28.65
4	209	114	2.19	28.96
Pure Cu	437	430	3.20	17.54
Pure Al	184	179	1.88	28.53

## Data Availability

Data sharing is not applicable.
